# Molecular science *vs*. molecular medicine

**DOI:** 10.1093/nsr/nwz181

**Published:** 2020-02-04

**Authors:** Weihong Tan, Jianhui Jiang, Chaoyong Yang

**Affiliations:** 1 Institute of Molecular Medicine (IMM), State Key Laboratory of Oncogenes and Related Genes, Renji Hospital, Shanghai Jiao Tong University School of Medicine, and College of Chemistry and Chemical Engineering, Shanghai Jiao Tong University, China; 2 Molecular Science and Biomedicine Laboratory (MBL), State Key Laboratory of Chemo/Biosensing and Chemometrics, College of Chemistry and Chemical Engineering, College of Biology, Aptamer Engineering Center of Hunan Province, Hunan University, China; 3 Institute of Cancer and Basic Medicine (IBMC), Chinese Academy of Sciences; The Cancer Hospital of the University of Chinese Academy of Sciences, China; 4 Department of Chemical Biology, College of Chemistry and Chemical Engineering, Xiamen University, China

Molecular medicine aims to understand the molecular mechanisms underlying disease pathogenesis in order to develop suitable diagnostic strategies and disease interventions. From this perspective, chemists can and have to play a pivotal role in the field of molecular medicine as chemists are molecular scientists. In 1949, the internationally renowned chemist Linus Pauling published his seminal work entitled ‘Sickle cell anemia, a molecular disease’, in *Science*, laying the groundwork for the field of molecular medicine. Over the past 70 years, chemistry has been one of the major forces pushing the field of molecular medicine to grow and evolve. Today, many important contributions made by chemists can be found in different subfields of molecular medicine, including molecular imaging, molecular diagnosis, molecular drug design and gene editing, *et al*. As in other areas of science and technology development, molecular medicine is thriving with active contributions of chemists and other molecular scientists.

This special topic features biomedical scientists, physicians and chemists, mostly from Shanghai Jiao Tong University, to showcase the influence of chemistry on the field of molecular medicine. In their review, Guo-Qiang Chen *et al.* summarize some recent progress in applying chemical biology to explore the molecular mechanisms of carcinogenesis, outlining several new strategies for chemistry to probe cellular activities. Ying-xuan Chen and Jing-Yuan Fang *et al.* discuss recent findings related to the crosstalk between microbiota and epigenetic modifications in colorectal cancer. Bing Su *et al.* review Sin1, a key adaptor molecule involved in the regulation and function of the mammalian target of rapamycin (mTOR) signaling pathway. Xinyuan Zhu and Deyue Yan *et al.* introduce recent advances in supramolecular designs of nanoscale drug delivery systems. In the perspective article by Xiawei Wei and Yuquan Wei *et al.*, opportunities and challenges in the use of nanoparticles for nucleic acid therapeutics are discussed. Guangjun Nie and Yuliang Zhao *et al.* review some recent trends in nanomedicine design and further discuss challenges and opportunities in the development of next-generation nanomedicines. The research highlight article by Chaoyong Yang *et al.* reports a recent finding tracking the levels of circulating exosomal PD-L1 may help to predict the response of patients and identify the possible reasons for success or failure of anti-PD-1 therapy. An interview with Feng Shao and Weihong Tan is also included in this special topic. They share their personal views of the impact of chemistry on the biomedical sciences, in addition to their opinions on biomedical research methods and career development.

To effectively tackle challenging problems in molecular medicine, interdisciplinary approaches and collaborations are essential. We hope this special topic will inspire more researchers from different disciplines of science, engineering, pharmacy and medicine, as well as commercial companies, to join in the exciting field of molecular medicine.

Finally, we would like to take this opportunity to express our gratitude to all the authors, reviewers, and the NSR editorial staff for their efforts in making this special topic possible.

